# Aqueous humor polymerase chain reaction in uveitis – utility and safety

**DOI:** 10.1186/s12886-016-0369-z

**Published:** 2016-10-28

**Authors:** Argyrios Chronopoulos, Daniel Roquelaure, Georges Souteyrand, Jörg Dieter Seebach, James Scott Schutz, Gabriele Thumann

**Affiliations:** 1 Department of Ophthalmology, University Hospitals and School of Medicine, Geneva, Switzerland; 2 Division of Immunology and Allergy, University Hospitals and School of Medicine, Geneva, Switzerland

**Keywords:** Uveitis, Anterior uveitis, Posterior uveitis, Anterior chamber paracentesis, Polymerase chain reaction (PCR)

## Abstract

**Background:**

To study the value and safety of aqueous humor polymerase chain reaction (PCR) analysis for Herpes simplex, varicella zoster, cytomegalovirus, Epstein-Barr virus and Toxoplasma gondii in patients with uveitis.

**Methods:**

Records of 45 consecutive patients with anterior and posterior uveitis who underwent AC paracentesis with PCR were reviewed. The main outcome measure was frequency of PCR positivity. Secondary outcomes were alteration of treatment, safety of paracentesis, and correlation of keratitic precipitates with PCR positivity,

**Results:**

The overall PCR positivity was 48.9 % (22/45). Therapy was changed because of the PCR results in 14/45 patients (37.7 %). One patient experienced a paracentesis related complication (1/45, 2.2 %) without long-term sequelae.

**Conclusion:**

Aqueous PCR altered the diagnosis and treatment in over a third of our patients and was relatively safe. Aqueous PCR should be considered for uveitis of atypical clinical appearance, recurrent severe uveitis of uncertain etiology, and therapy refractory cases.

## Background

Rapid analysis of aqueous humor by polymerase chain reaction (PCR) in uveitis can be very helpful in leading to a specific diagnosis of infectious etiology and efficient treatment [1–7]. However, aqueous humor PCR analysis is not routine and remains controversial because of a low proportion of therapeutic change based on the PCR results in some studies, invasiveness of paracentesis bearing potential complications, and false negatives and positives [5–7]. On these grounds recent reports even question its utility and discourage its practice in anterior uveitis [5, 8]. Recent reports discourage aqueous PCR for the diagnosis of infectious uveitis or favour PCR of vitreous over aqueous [5, 9, 10].

Although in most cases the working diagnosis of uveitis is based on history and clinical signs, supported by routine blood tests and radiological examinations, diagnostic error can cause increased ocular morbidity and establishing a definitive etiology often can be challenging. Quick identification of infectious etiology is particularly important in management of posterior uveitis because fundus lesions caused by viruses, bacteria, fungi or parasites cannot always be clearly distinguished, especially in the presence of opaque vitreous. Also, the significant proportion of uveitis characterized as idiopathic might be reduced if PCR testing were performed [11].

In this study we report the results of aqueous PCR analysis for infectious agents and the rate of treatment change based on the PCR results in patients with anterior, posterior, intermediate uveitis, and panuveitis, as well as the complication rate of paracentesis. We also analyze the association of keratic precipitates with PCR positivity and suggest guidelines for performing a safe anterior chamber paracentesis at the slitlamp.

## Methods

This study was carried out following institutional guidelines and ethical standards of the 1964 Declaration of Helsinki and its later amendments and was approved by the local ethical committee *of the Geneva University Hospitals and School of Medicine (Commission cantonale d'éthique de la recherche CCER)* (No. 15-188). A retrospective review of consecutive patients with anterior, posterior, intermediate uveitis and panuveitis who underwent aqueous PCR analysis was performed by cross-referencing the PCR test list of the Institute for Immunology and Microbiology with the clinical database of the Department of Ophthalmology from January 1, 2011 to March 1, 2015 and yielded 45 uveitis patients (45 eyes).

The 45 patients’ records were reviewed for initial working diagnosis, disease course, signs and degree of ocular inflammation prior to paracentesis, clinical rationale for paracentesis, intraocular pressure, PCR results, and paracentesis complications. The grading and classification of uveitis followed the Standardization of Uveitis Nomenclature (SUN) recommendations [12]. In all cases, diagnostic AC paracentesis was performed at the slit lamp following topical anesthesia, disinfection with povidone*-*iodine, and placement of a sterile lid speculum. A 27- or 30-gauge needle on a 3 ml syringe was used to extract about 0.2 ml of aqueous humor. Semi-quantitative real-time PCR (rt-PCR) was performed within 24 h of sample collection for Herpes simplex (HSV1/2), varicella zoster virus (VZV), cytomegalovirus (CMV), Epstein-Barr virus (EBV) and Toxoplasma gondii DNA. Nucleic acid from aqueous humor was extracted using the EasyMag Nuclisens® (bioMérieux Inc., Durham, NC, USA) (100 μl eluted in 25 μl respectively) and used for rt-PCR with an in-house validated method on a StepOne Plus ABI® machine (Thermo Fisher Scientific Inc, Waltham, MA USA). A signal detected during the first 40 cycles revealed the presence of foreign DNA. The primers and probes sequences used for the detection of HSV 1/2, VZV, CMV, EBV and Toxoplasma gondii DNA are given in Table 1.

**Table 1:** PCR primers and probes sequences

HSV	Forward	5’- CCGTCAGCACCTTCATCGA -3’
	Reverse	5’-CGCTGGACCTCCGTGTAGTC -3’
	Probe	5’-CCACGAGATCAAGGACAGCGGCC-3’
VZV	Forward	5’- CGG CAT GGC CCG TCT AT -3’
	Reverse	5’-TCG CGT GCT GCG GC -3’
	Probe	5’-ATT CAG CAA TGG AAA CAC ACG ACG CC-3’
Toxo	Forward	5'- AGA GAC ACC GGA ATG CGA TCT - 3'
	Reverse	5'- CCC TCT TCT CCA CTC TTC AAT TCT – 3'
	Probe	5' -ACG CTT TCC TCG TGG TGA TGG CG -3'
EBV	Forward	5'- CGG AAG CCC TCT GGA CTT C -3’
	Reverse	5'- CCC TGT TTA TCC GAT GGA ATG -3’
	Probe	5'- TGT ACA CGC ACG AGA AAT GCG CC -3’
CMV	Forward	5'- GATCCGCTGACGCGTTTG-3’
	Reverse	5'- GCCGCCAGTCGTAACGAT-3’
	Probe	5'- TCATCGATCGGCGGATCACCAC -3’

The presence of foreign DNA was assessed by semi-quantitative rt-PCR. Table 1 demonstrates the PCR primers and probes sequences for HSV, VZV, CMV, EBV and Toxoplasma gondii that were used

The primary outcome measure was the frequency of PCR positivity. Secondary outcomes were change in treatment based on the PCR results, safety profile of diagnostic AC paracentesis, and correlation between keratic precipitates (KP) and positive PCR results.

Descriptive statistics were applied to continuous data while categorical data were assessed using IBM SPSS statistical software version 20 (SPSS, Inc, Chicago, Illinois, USA).

## Results

Demographics of the 45 patients are presented in Table 2. Five of the 45 patients underwent repeated paracentesis (average 2.6) because of a negative PCR result in patients strongly suspected of having infectious uveitis, so there were 53 samples in total. Mean age was 43.6 ± 18 years with a female to male ratio of 1:1.6. Indications for paracentesis were: 6.7 % (3/45) recurrent anterior uveitis, 13.3 % (6/45) *hypertensive anterior uveitis (Posner Schlossman syndrome) (PSS)*, 13.3 % (6/45) anterior uveitis of suspected viral/microbial etiology based on clinical features including granulomatous KP and iris atrophy, 4.5 % 2/45) *anterior uveitis with suspicion of Fuchs heterochromic uveitis syndrome (FUS)*, 44.4 % (20/45) suspected viral/microbial posterior uveitis, 15.6 % (7/45) panuveitis, and 2.2 % (1/45) intermediate uveitis of uncertain etiology. Table 3 outlines the overall results and subgroup analysis for PCR positivity. *In 21 of the 45 patients (six anterior uveitis, 11 posterior uveitis, one intermediate uveitis, three panuveitis) sampling occurred without previous anti-inflammatory, antiviral treatment. Seven patients (three anterior uveitis and four posterior uveitis) were already under anti-inflammatory therapy at the time of sampling which did not change. Five patients (three anterior uveitis and two posterior uveitis) were already on anti-infectious before sampling, which changed following the result of PCR analysis. Three posterior uveitis, four panuveitis and five anterior uveitis had no antiinfectious therapy prior to PCR testing but then received it after PCR analysis.*

**Table 2:** Patient demographics

	Age	Working Diagnosis	PCR	CMV	HSV	VZV	EBV	Toxoplasma	Treatment altered
1	22	Uveitis posterior, Adamantiadis-Behçet	neg						
2	47	Toxoplasma retinitis	pos*					+	yes
3	28	Uveitis anterior, Posner Schlossman	pos*^,^**			+	(+)		
4	30	Uveitis posterior, ARN	pos			+			yes
5	54	Recurrent granulomatous anterior uveitis	neg						yes
6	32	Recurrent nongranulomatous anterior uveitis	neg						
7	78	Recurrent granulomatous anterior uveitis with iris atrophy	pos			+			yes
8	14	Toxoplasma retinitis	pos					+	yes
9	31	Toxoplasma retinitis	pos					+	yes
10	52	Posterior uveitis, Birdshot	neg						
11	23	Uveitis posterior, Adamantiadis-Behçet	neg						
12	47	Anterior uveitis, suspicion on FUS	pos		+				
13	61	Posterior uveitis, ARN	pos*			+			
14	26	Posterior uveitis, Adamantiadis-Behçet	neg						
15	30	Posterior uveitis, Adamantiadis-Behçet	neg						
16	22	Acute anterior uveitis	neg						
17	47	Acute anterior uveitis	neg						
18	78	Intermediate uveitis	neg						
19	41	Anterior uveitis	neg						
20	43	Panuveitis granulomatous	pos					+	yes
21	20	Panuveitis	neg						
22	58	Panuveitis, Adamantiadis-Behçet	pos					+	yes
23	29	Anterior uveitis, nongranulomatous, *hypertensive*	neg						yes
24	73	Posterior uveitis, ARN	pos*				+		yes
25	27	Posterior uveitis	pos**		(+)			+	
26	19	Panuveitis	pos					+	yes
27	23	Panuveitis	pos**				(+)	+	yes
28	54	Anterior uveitis, Posner Schlossman	pos	+					yes
29	36	Anterior uveitis, Posner Schlossman	neg*						yes
30	45	Anterior uveitis, Posner Schlossman	pos	+					yes
31	65	Anterior uveitis, suspicion FUS	pos		+				yes
32	28	Posterior uveitis, vasculitis	neg						
33	39	Posterior uveitis, VKH	neg						
34	26	Posterior uveitis	neg						
35	68	Posterior uveitis	neg						
36	68	Posterior uveitis, Toxoplasma retinitis	pos					+	
37	58	Panuveitis, occlusive vasculitis	neg						
38	22	Posterior uveitis, vasculitis	neg						
39	39	Panuveitis	neg						
40	81	Anterior uveitis	pos		+				
41	19	Posterior uveitis	neg						
42	69	Recurrent anterior uveitis, Posner Schlossman	pos		+				yes
43	38	Acute anterior uveitis	neg						
44	49	Recurrent anterior uveitis, Posner Schlossman	pos	+					
45	43	Posterior uveitis	pos					+	

Demonstrates the patient cohort demographics which included 28 men and 17 women with a mean age of 43 years. Aqueous humor of 22 patients was PCR positive for either CMV, HSV, VZV, EBV or Toxoplasma gondii. 5 patients were tested multiple times, indicated by a *. 3 patients were PCR positive for two infectious agents, indicated by a **. Potentially false positive results are noted in parenthesis

**Table 3:** Overall and subgroup PCR positivity

Patient PCR Positivity	22 out of 45 (48.9 %) (95 % CI 46 %-51 %)	
Sample Positivity	22 out of 53 (41.5 %)	
Diagnosis	#	PCR positivity
Anterior uveitis	17/45 (37.8 %)	9/17 (52.9 %)
Posterior uveitis	20/45 (44.4 %)	9/20 (45 %)
Panuveitis	7/45 (15.6 %)	4/7 (57.1 %)
Intermediate uveitis	1/45 (2.2 %)	0 %

Overall and subgroup PCR positivity according to uveitis diagnoses

### Overall PCR positivity

In total, 22/53 samples were positive for presence of foreign DNA (41.5 %). Two samples from one patient drawn within 48 h were positive for the same pathogen and counted only once in the analysis. 7/45 patients (15.6 %) were immunosuppressed at the time of paracentesis, 5 with Adamantiadis-Behçet vasculitis and already on corticosteroids, 1 suspected to have Birdshot chorioretinopathy, and 1 with bilateral posterior uveitis and occlusive vasculitis of unknown etiology.

### Patients with anterior uveitis

Of the 17 patients with anterior uveitis, 9 were PCR positive: 4 positive for HSV (2 suspicious for FUS, 1 PSS, and 1 acute granulomatous anterior uveitis), 3 positive for CMV (clinically with PSS), and 2 positive for VZV (1 PSS with double positivity for EBV and 1 recurrent granulomatous anterior uveitis with iris atrophy). The remaining 8 patients were negative: 5 with acute anterior uveitis and 3 with recurrent anterior uveitis.

### Patients with posterior uveitis

Of the 20 patients with posterior uveitis, 9 were PCR positive: 5 clinically diagnosed with toxoplasmosis also positive for *Toxoplasma gondii* and 1 positive also for HSV, 2 positive for VZV with clinical acute retinal necrosis (ARN), and 1 positive for EBV also with ARN. The remaining 11 patients were PCR negative and were finally diagnosed as: 4 Adamantiadis-Behçet's retinitis, 1 Birdshot chorioretinopathy, 1 Vogt-Koyanagi-Harada (VKH) syndrome, 2 vasculitis of undetermined etiology, and 3 undetermined posterior uveitis.

### Patients with panuveitis or intermediate uveitis

Of the 7 patients with panuveitis, 3 were PCR positive for Toxoplasma gondii alone, one for Toxoplasma gondii and EBV, 3 PCR negative. The one patient with intermediate uveitis was PCR negative.

### Treatment change based on PCR

Treatment was changed based on PCR results in 17/45 patients (37.7 %): 9/17 anterior uveitis (53 %), 4/20 posterior uveitis (20 %), and 4/7 panuveitis patients (57 %), all of whom responded favorably. One patient with recurrent VZV associated anterior uveitis was placed on valaciclovir; two patients with acute retinal necrosis (one VZV and one EBV associated) were placed on ganciclovir; 7 patients positive for Toxoplasma gondii were placed on antibiotics; *3 patients with PSS (two CMV positive and one HSV positive) received systemic valaciclovir replaced later on by topical 2 % ganciclovir solution and systemic valaciclovir respectively and one presumed FUS patient finally tested positive for HSV1 and was placed on systemic acyclovir*. In 3 anterior uveitis patients after negative PCR with repeated testing, acyclovir was discontinued and replaced with topical corticosteroids.

### Association of KP with PCR positivity

The association of KP with infectious uveitis is confirmed; 16/21 KP positive eyes had a positive PCR (76.2 %) whereas only 6/24 KP negative eyes had a positive PCR (25 %) (Table 4). Sensitivity and specificity were 72.7 % and 78.3 % respectively (*p* = 0.002, *Chi square* = 9.8, power = 0.9). In the subgroup analysis on HSV, VZV, CMV, EBV and Toxoplasma gondii, the percentage of KP presence with PCR positivity was 80 %, 75 %, 100 %, 66.7 % and 70 % respectively (Table 4).

**Table 4:** Correlation between keratitic precipitates (KP) and PCR results

PCR +	KP +	KP -	% PCR positive with KP
HSV	4	1	80
CMV	3	1	100
VZV	3	0	75
EBV	2	1	66.7
Toxoplasma	7	1	70

Overall and subgroup PCR positivity in correlation with the presence or absence of KPs and the infectious agents detected. 16/21 KP positive eyes had a positive PCR whereas only 6/24 KP negative eyes had a positive PCR

### Safety profile

Only one patient developed what appeared to be a paracentesis-related complication, increased anterior chamber inflammation (2.2 %) and was hospitalized for 24 h without long-term sequelae.

## Discussion

Diagnostic uncertainty and dilemmas are common in the management of uveitis. In this study, aqueous PCR analysis was of considerable diagnostic value with 48.9 % of patients positive for infectious DNA leading to a change in diagnosis and treatment in over a third of the patients (37.7 %), all of whom responded favorably to the new treatment. It is likely that with a larger test battery for foreign DNA, the yield of detection of specific infectious pathogens would be even greater. PCR from ocular fluids has a very high reliability with a very low false-positive rate [13–16]. False negative results are more difficult to verify as there is no gold standard for the accuracy of PCR and viral or Toxoplasma gondii cultures are rarely performed [17].

Aqueous PCR analysis in uveitis has been previously reported [5–7, 15, 18, 19]. Harper et al. reported a 20 % change in the management of patients with posterior uveitis following PCR results [6] and Rothova et al. reported foreign DNA in 29 % of posterior uveitis patients with a change in management in 24 %. Anwar et al. reported positivity for foreign DNA in only 13 % of 53 anterior uveitis patients leading to a change in management in 24 %, only 3 % of the total study group, and expressed doubts about its usefulness [5, 7].

At present, with limited PCR testing, infectious causes represent around 20-30 % of all uveitis with Herpes viruses the most common cause of anterior uveitis in Western countries [20–23]. Our subgroup analysis supports this finding. A viral etiology was particularly frequent in *hypertensive* anterior uveitis with 85.7 % (6/7) of patients positive for CMV, VZV or HSV. *The presence of an infectious agent was also confirmed in two cases of suspected FUS found to be positive for the Herpes virus genome and who were treated accordingly*. In our posterior uveitis patients, we observed a high prevalence of Toxoplasma gondii (10/45, 22.2 %) and lower rates of CMV, HSV and VZV positivity compared to Asian and US studies which reported CMV or CMV and HSV as leading infectious causes followed by Toxoplasma gondii [6, 19]. Previous systematic surveys on uveitis from Western Europe confirm Toxoplasmosis as the most common cause of infectious posterior uveitis which could be related to local customs of consumption of very rare meat and raw chopped steak [23–25]. Aqueous PCR testing for Toxoplasma gondii seems to be reliable and not influenced by the interval between symptom onset and paracentesis or host immune status but rather by the total size of the retinal lesions [18, 26]. *Furthermore aqueous PCR testing can be particularly useful for excluding an infectious aetiology in immunocompromised or older patients as the clinical presentation can be atypical and misleading* [15, 26–29], *in severe anterior uveitis cases with no fundal view, in severe presumed HLA-B27 uveitis* [30] *and in cases of presumed infectious posterior uveitis with atypical and sight-threatening inflammation where a rapid and sensitive diagnosis from prompt sampling for PCR analysis is crucial*.

Three patients, one PSS, one posterior uveitis, and one panuveitis, were double positive, for VZV and EBV, EBV and Toxoplasma gondii, and HSV and Toxoplasma gondii, respectively, with weak signals for both EBV and Toxoplasma gondii. In these double positive cases, therapy was influenced by clinical criteria with close follow-up. Double PCR positivity, which can lead to diagnostic challenges, has been previously reported in immunosuppressed or otherwise immunocompromised patients and may be caused by dormant latent viruses or parasites activated secondary to acute infectious uveitis [6, 31–33]. In the particular case of EBV, a major limitation is possible cross reactivity with B lymphocytes containing EBV genome in a latent phase, which can lead to false positive results [13]. *This phenomenon of dual positivity has been observed in previous studies and in particular in immunocompromised patients; in those cases PCR testing of ocular fluids helps substantially, testing for multiple pathogens before taking the final decision for treatment based on clinical appearance as the phenotype of the intraocular inflammation can be misleading in such cases*.

The complication rate of anterior chamber paracentesis in uveitis is low as corroborated in our study [34]. Paracentesis performed at the slit lamp has a low complication rate if: 1) collaborative adult patients with a deep anterior chamber are selected; 2) the eye is properly prepared; 3) a speculum is used; and 4) careful follow-up is possible. Otherwise, paracentesis supine using an operating microscope and appropriate sedation or anesthesia may be indicated. In our experience, it is essential that the hand holding the syringe be braced securely on the patient’s face and not on the frame of the slit lamp and that the patient’s head rests firmly on the head and chin rests to reduce head movement. *Informed consent is required including as potential complications endophthalmitis, corneal abscess, hyphema, conversion to vitreous sampling, and trauma to the cornea, iris, lens, and posterior segment*.

Our study has several limitations. First, it was performed in a tertiary care center including uveitis patients in whom there was a strong suspicion for infectious etiology creating a possible referral and selection bias. Furthermore, different geographic locations have different frequency/prevalence of uveitis pathogens and PCR detection thresholds may vary accordingly between studies [5, 19, 35]. While our study demonstrates a 48.9 % positivity for foreign DNA, the use of antivirals prior to paracentesis may have reduced the virus load below detectable levels in some patients. Finally, although FUS has been strongly associated with rubella virus, it was not tested in our study [36]. *One further pathogen that was not tested with PCR but which merits consideration in relevant clinical settings is Treponema pallidum* [37]. *Although Treponema pallidum infections were considered almost eradicated in developed countries, epidemiologic studies confirm steady increase of primary syphilis infections with a considerable percentage of secondary, ocular manifestations requiring prompt diagnosis and treatment* [38, 39].

## Conclusion

Aqueous PCR analysis is easy to perform, safe, and valuable, establishing a correct etiologic diagnosis and change in treatment in about a third of selected uveitis cases. We consider aqueous PCR most useful for uveitis with atypical clinical appearance, recurrent severe uveitis of uncertain etiology, and treatment-resistant cases. *In suspected infectious uveitis where retinal examination may be limited by vitreous opacification prompt intraocular fluid sampling is necessary and important in reaching a preliminary diagnosis and administering therapy. Regardless, empiric treatment should be initiated based on the clinical picture in severe, sight-threatening uveitis cases with no delay; this can be further adjusted depending on PCR results; negative results can be useful in excluding infectious etiology*. In the future, a larger battery of PCR tests for infectious agents will probably become routine for diagnosing infectious uveitis with even better accuracy.

## Abbreviations

ARN: Acute retinal necrosis
CMV: Cytomegalovirus
EBV: Epstein-bar virus
FUS: Fuchs uveitis syndrome
HSV: Herpes simplex virus
Pos/neg: Positive/negative PCR
PSS: Posner-Schlossman syndrome
VKH: Vogt-Koyanagi-Harada-Syndrom
VZV: Varicella zoster virus
